# Armed Conflict and Penetrating Traumatic Brain Injury in Children in the Gaza Strip

**DOI:** 10.1001/jamanetworkopen.2026.13094

**Published:** 2026-05-15

**Authors:** Mohammed Asfa, Joakim Stray Andreassen, Abdul Basit Khan, Geir Stray Andreassen, Mohammed A. K. Matar, Alaa A. A. Alaqad, Ihab Z. D. Nassar, Moath A. J. Albhnasawi, Øyvind Olav Salvesen, Sasha Gulati, Nidal Abuhadrous

**Affiliations:** 1 Department of Neurosurgery, European Gaza Hospital, Al-Fukhari, Gaza Strip, Palestine; 2 Department of Neurosurgery, St Olavs University Hospital, Trondheim, Norway; 3 Department of Circulation and Medical Imaging, Norwegian University of Science and Technology (NTNU), Trondheim, Norway; 4 Department of Health Sciences, University of Oklahoma, Oklahoma City; 5 Norwegian Aid Committee (NORWAC), Oslo, Norway; 6 Department of Orthopaedics, Oslo University Hospital, Oslo, Norway; 7 Department of Public Health and Nursing, Norwegian University of Science and Technology (NTNU), Trondheim, Norway; 8 Department of Neuromedicine and Movement Science, Norwegian University of Science and Technology (NTNU), Trondheim, Norway

## Abstract

This cohort study assesses functional outcomes at hospital discharge among children and adolescents who sustained penetrating traumatic brain injury during armed conflict in the Gaza Strip.

## Introduction

Penetrating traumatic brain injury (TBI), characterized by a breach of the dura mater caused by a projectile or fragment, represents one of the most fatal war-related injuries.^1^ Survivors frequently endure neurological impairments alongside long-term psychosocial challenges.^2^ Evidence on the characterization, management, and outcomes of pediatric penetrating TBI in conflict settings remains scarce.^3,4^ This gap is concerning given the scale of recent violence; according to the Palestinian Ministry of Health, over 20 000 children were killed during the recent Gaza-Israel conflict.^5^ The aim of this study was to assess functional outcomes at hospital discharge among children and adolescents who sustained penetrating TBI during armed conflict in the Gaza Strip.

## Methods

This cohort study was conducted at Department of Neurosurgery of the European Gaza Hospital and Nasser Medical Complex from January 1, 2025, to September 30, 2025. Ethics approval was obtained from the Palestinian Health Research Council with a waiver of informed consent. The study adhered to the STROBE reporting guidelines for cohort studies.^6^ Patients aged 18 years or younger admitted with penetrating TBI were registered using online spreadsheets. Diagnosis and mechanism of injury were determined by neurosurgeons using history, clinical examination, and computed tomography imaging.

Severity of TBI was assessed using the Glasgow Coma Scale (GCS) at admission and according to standard definitions; a score of 14 to 15 was classified as mild TBI, 9 to 13 as moderate TBI, and 8 or lower as severe TBI.^7^ Patients who were intubated before hospital admission or before neurosurgical evaluation did not have a preintubation GCS recorded and were, therefore, categorized as a separate group. The primary outcome was assessed by Glasgow Outcome Scale at discharge, which categorizes recovery into 5 levels: death, vegetative state, severe disability, moderate disability, and good recovery. Moderate disability and good recovery were classified as favorable outcomes.

Continuous variables were summarized as medians (IQRs), and categorical variables were summarized as frequencies (percentages). Comparisons were performed using the χ^2^ test, with 2-sided *P* < .05 considered statistically significant for comparisons. Missing data were excluded without imputation. Analyses were performed using R statistical software version 4.4.0 (R Project for Statistical Computing).

## Results

Among 719 patients with penetrating TBI during the 273-day study period, 321 (45%) were aged 18 years or younger (median [IQR] age, 13.0 [8.0-16.0] years; 229 male [72%]), and the average daily admission rate for pediatric penetrating TBI was 1.18 patients. Demographic characteristics, injury mechanisms, radiological findings, and neurosurgical interventions are summarized in the Table.

**Table.** Demographics, Injury Mechanism, Radiological Findings, and Treatment of Pediatric Patients With Penetrating Traumatic Brain Injury

Characteristic	Patients, No. (%)		
	Overall (N = 321 [100%])	Gunshot wound (n = 29 [9%])	Shrapnel (n = 292 [91%])
Age, median (IQR), y	13.0 (8.0-16.0)	13.0 (9.0-16.0)	13.0 (8.0-15.5)
Sex			
Male	229 (72)	21 (72)	208 (71)
Female	91 (28)	8 (28)	83 (29)
Unknown	1 (<1)	0	1 (<1)
GCS score on hospital admission, median (IQR)	14 (10-15)	15 (10-15)	14 (10-15)
Severity of traumatic brain injury			
Mild (GCS score 14-15)	110 (36)	10 (34)	100 (36)
Moderate (GCS score 9-13)	61 (20)	5 (17)	56 (20)
Severe (GCS score ≤8)	38 (12)	2 (7)	36 (13)
Intubation before neurosurgical assessment	100 (32)	12 (41)	88 (31)
Unknown	12	0	12
Pupils on arrival			
Unilateral dilatation	26 (8)	5 (17)	21 (7)
Bilateral dilatation	20 (6)	1 (3)	19 (7)
Unilateral or bilateral ruptured globe	3 (1)	0	3 (1)
Brain extrusion	68 (21)	9 (31)	59 (20)
Radiological findings			
Compartment			
Supratentorial injury	295 (92)	26 (90)	269 (92)
Infratentorial injury	7 (2)	1 (3)	6 (2)
Both compartments	19 (6)	2 (7)	17 (6)
Retained brain shrapnel	97 (30)	8 (28)	89 (30)
Unilateral injury	195 (61)	22 (76)	173 (59)
Bifrontal injury	23 (7)	0 (0)	23 (8)
Transventricular injury	63 (20)	7 (24)	56 (19)
Surgery	53 (17)	12 (41)	41 (14)
Craniectomy	41 (13)	7 (24)	34 (12)
Evacuation of mass lesion	5 (2)	0	5 (2)
External ventricular drain	4 (1)	0	4 (1)
Bullet or shrapnel extraction	7 (2)	5 (17)	2 (1)
Extensive wound debridement	2 (1)	0	2 (1)
Bedside debridement and closure	215 (67)	14 (48)	201 (69)
Length of stay, median (IQR), d	5 (3-10)	4 (2-11)	5 (3-10)

Abbreviation: GCS, Glasgow Coma Scale.

Injury mechanisms included shrapnel (292 patients [91%]) and gunshot wounds (29 patients [9%]). At admission, 110 patients (36%) had mild TBI, 61 (20%) had moderate TBI, 38 (12%) had severe TBI, and 100 patients (32%) were intubated before neurosurgical assessment; 53 patients (17%) underwent surgery.

Favorable outcomes were observed in 154 patients (49%), and 94 patients (30%) died (Figure). The outcome at discharge was unknown for 8 patients (2%). Favorable outcomes were observed in 90 patients (83%) with mild TBI, compared with 31 patients (53%) with moderate TBI, 10 patients (26%) with severe TBI, and 21 patients (21%) who were intubated before neurosurgical assessment.

**Figure.**
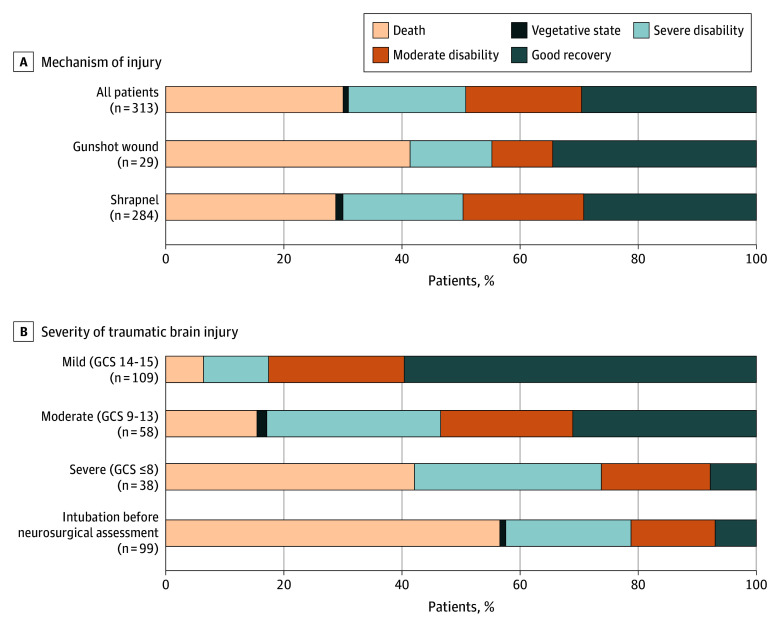
Bar Graphs of Glasgow Outcome Scale Outcomes at Discharge Graphs show outcomes according to injury mechanism (A) and severity of traumatic brain injury (B). GCS indicate Glasgow Coma Scale.

## Discussion

In this cohort study of children and adolescents with penetrating TBI, there was substantial morbidity and mortality. To our knowledge, this study represents the largest single-center dataset from a contemporary war zone of pediatric patients with penetrating TBI.^8^

A prior observational study^3^ identified 392 pediatric patients with penetrating TBI between 2 war zones over a longer time (Iraq and Afghanistan, 2004-2012). Separate reports on penetrating TBI during the invasion of Iraq and Afghanistan (2003-2011) identified that 51 of 813 patients (6%) were children.^4^ In our cohort, 45% of patients with penetrating TBI were aged 18 years or younger, confirming that children are disproportionately affected by penetrating TBI and at even higher rates than prior conflicts. Unlike a similar report,^3^ in which 51% of pediatric penetrating TBI cases underwent surgery, only 17% received operative care in the current study. The high volume of trauma patients, compounded by ongoing bombings in the immediate vicinity of the hospital, severely overstretched surgical facilities, necessitating strict prioritization limiting neurosurgical care. Critical shortages of neurosurgical equipment and basic surgical supplies further constrained treatment capacity. To improve outcomes in conflict zones, reliable access to these supplies, adequate imaging capacity, anesthesia services, intensive care unit capacity, and sufficient operating room availability are essential. Our findings reaffirm the obligations under international humanitarian law to protect children and prevent acts of violence against them. Upholding these principles is essential to reduce the burden of preventable pediatric neurotrauma in conflict zones.

Study limitations include absence of long-term functional outcome data, lack of information on prehospital fatalities, incomplete data on preintubation GCS, physiological parameters, and extracranial injuries. Another limitation is that we do not know the preconflict incidence of pediatric penetrating TBI, nor do we have preconflict case data, because systematic data collection began during the conflict period. Despite these limitations, this study provides insight into pediatric patients with penetrating TBI, a population seldom represented in the literature. The data reflect clinical practice in a resource-limited conflict setting and provide evidence of injury patterns and outcomes in this patient cohort.

## Notes

Supplement. Data Sharing Statement
