# Supplementary material for: Armed Conflict and Penetrating Traumatic Brain Injury in Children in the Gaza Strip
Source: JAMA Netw Open. 2026 May 15;9(5):e2613094. doi: 10.1001/jamanetworkopen.2026.13094 (PMC13179554; doi:10.1001/jamanetworkopen.2026.13094)
Supplement: Supplement. — Data Sharing Statement [file jamanetwopen-e2613094-s001.pdf]

Data Sharing Statement

Asfa. Armed Conflict and Penetrating Traumatic Brain Injury in Children in the Gaza Strip. *JAMA Netw Open*. Published May 15, 2026. doi:10.1001/jamanetworkopen.2026.13094

Data
Data available: Yes
Data types: Deidentified participant data
How to access data: Upon request, the deidentified participant data can be made available, contact corresponding author at Asfa1991@gmail.com
When available: With publication

Supporting Documents
Document types: Statistical/analytic code
How to access documents: Statistical code with R scripts can be made available upon request by contacting joakim.s.andreassen@ntnu.no
When available: With publication

Additional Information
Who can access the data: Will be considered for anyone requesting the data
Types of analyses: R scripts
Mechanisms of data availability: After approval of a proposal
